# Integración de tecnologías digitales en la enseñanza de competencias interculturales: mapeo sistemático de la literatura

**DOI:** 10.12688/f1000research.167364.2

**Published:** 2025-10-14

**Authors:** Andrea Basantes-Andrade, Gabriela Bastidas-Amador, Claudia Ruiz-Chagna, Marlen Congo-Cervantes, Gabriela Quintana-Andrade

**Affiliations:** 1 Network Science Research Group (eCIER), Universidad Técnica del Norte, Ibarra, Imbabura, 100105, Ecuador; 2 Universidad Técnica del Norte, Ibarra, Imbabura, 100105, Ecuador

**Keywords:** intercultural competences, intercultural education, digital education, digital technologies, higher education, COIL, systematic mapping, systematic review

## Abstract

**Background:**

The development of intercultural competences mediated by digital technologies has gained prominence in higher education, driven by academic internationalization and the advancement of virtual learning environments. However, questions remain regarding the most frequently studied competence dimensions, the geographic regions involved, and the methodologies employed.

**Methods:**

A systematic literature mapping was conducted following the PRISMA 2020 guidelines to analyze scientific production from 2005 to 2024, sourced from Scopus, Web of Science, and SciELO. Inclusion criteria focused on peer-reviewed, open-access articles, in English or Spanish, addressing the integration of digital technologies in the teaching of intercultural competence. After screening 176 records, 23 studies were included for analysis.

**Results:**

Scientific production was primarily concentrated in the Americas and Europe (78%), with Mexico (15%) and countries such as the United States, Spain, and Russia each accounting for 11%. The most frequent competence dimensions were adaptation and management (47.83%), intercultural knowledge (39.13%), communication skills (39.13%), and intercultural attitudes (39.13%). Competences such as cultural adaptability, intercultural awareness, and intercultural communication were the most frequently addressed, reflecting the need for flexibility and communication skills in multicultural contexts. Technologies like COIL (Collaborative Online International Learning) and immersive tools are emerging as effective strategies to promote intercultural learning, although areas such as innovative leadership (4.35%) and personal development (8.70%) remain underexplored. Temporally, there has been sustained growth in research since 2019, with peaks in 2021, 2022, and 2023.

**Conclusion:**

Digital technologies show significant potential for the development of intercultural competences, but their implementation requires addressing structural, pedagogical, and equity-related gaps. This study lays the groundwork for future research and policies aimed at enhancing inclusive and sustainable intercultural education in digital environments.

## Introduction

In an increasingly globalized world, the development of intercultural competences has become an essential component of higher education, as it enables students to operate effectively in diverse and multicultural environments ( Guillén-Yparrea & Ramírez-Montoya, 2023a; Kuffuor et al., 2024). These competences go beyond attitudes toward other groups; they encompass understanding and knowledge of different worldviews, as well as the ability to adapt behaviorally for effective interaction ( Schwarzenthal et al., 2019).

Intercultural competence is defined as the combination of knowledge about various cultures and sociocultural contexts, together with communication skills and key attitudes such as empathy, openness, and adaptability ( Nikiforova & Skvortsova, 2021; Rokos et al., 2023). Its development is crucial for promoting inclusion, strengthening social cohesion, improving employability, and facilitating collaboration in international settings ( Hei et al., 2019; Sierra-Huedo & Foucart, 2022). It is built upon three essential elements: intercultural communication, which enables meaningful expression and understanding of messages ( Gutiérrez-Santiuste & Ritacco-Real, 2024); intercultural sensitivity, which fosters empathy and the recognition of cultural differences ( Huang et al., 2024; Swartz et al., 2020); and intercultural responsibility, which involves an ethical commitment to solidarity, critical cooperation, and mutual respect ( Neubauer, 2022; Zhang, 2024). These elements are key for building more inclusive and collaborative societies.

Traditionally, pedagogical approaches have prioritized unidirectional models with few opportunities for intercultural interaction ( Mara, 2021; Nguyen et al., 2022). However, the advancement of digital technologies has transformed these educational paradigms, providing innovative tools for the teaching of intercultural competences. Virtual platforms, communication applications, and collaborative learning environments have proven effective in fostering awareness and interaction among students from diverse cultures, promoting experiential learning and the development of intercultural skills ( Guillén-Yparrea & Ramírez-Montoya, 2023a; Li et al., 2020; Nguyen et al., 2022).

Recent studies highlight that digitally based experiential teaching enhances student motivation and engagement and strengthens their ability to critically analyze their cultural identity in relation to others ( Han et al., 2022; Kholod et al., 2023; Li et al., 2020). Moreover, the use of immersive environments, such as the metaverse, has emerged as an innovative strategy for developing global competences, allowing interaction in simulated scenarios that replicate multicultural contexts ( Guillén-Yparrea & Ramírez-Montoya, 2023a). This experiential approach facilitates the practice of skills in a controlled and safe environment, promoting the development of empathy, adaptability, and communication skills—essential elements in a multicultural context ( Pei et al., 2023).

The growing interest in integrating digital technologies into the teaching of intercultural competences has driven advances in the field; however, the current literature still faces significant challenges. These include the scarcity of systematic studies that allow for generalization of results and assessment of the long-term effectiveness of these educational strategies ( Guillén-Yparrea & Ramírez-Montoya, 2023a; Klimova & Chen, 2024; Wang et al., 2024); the need to understand the impact of technology on the development of communication skills and the consolidation of inclusive attitudes in globalized contexts ( Melnyk & Koroban, 2024); and the analysis of their ethical and pedagogical implications, particularly regarding the use of immersive digital tools such as artificial intelligence and virtual reality in intercultural teaching ( Klimova & Chen, 2024).

In response to these gaps, the present study conducts a systematic literature mapping to analyze the integration of digital technologies in the teaching of intercultural competences. Articles published between 2005 and 2024 were examined, following the PRISMA protocol and selecting studies indexed in high-impact databases. This methodological approach ensures the validity and reliability of the findings, enabling the identification of trends, methodological approaches, and research gaps.

To guide the analysis, the following six research questions were formulated:

RQ1. In which countries and/or regions is research concentrated?

RQ2. What types of digital technologies are used in the teaching of intercultural competences?

RQ3. In which areas does research on the integration of digital technologies focus, such as communication skills, cultural attitudes, and technological adoption?

RQ4. Which intercultural competences are addressed?

RQ5. What are the limitations of the research?

RQ6. What are the future research directions regarding intercultural competences?

This study provides a comprehensive overview of advances in the teaching of intercultural competences through digital technologies, establishing a reference framework for designing future research and developing educational policies aimed at promoting inclusion and diversity in academic and professional contexts.

## Methods

This study presents a systematic literature mapping (SLM) aimed at analyzing the scientific production on the integration of digital technologies in the teaching of intercultural competences. This methodology provides a comprehensive overview of the available literature within a defined time frame, identifying trends, methodological approaches, and research gaps ( Kitchenham et al., 2009). Systematic literature mapping complements a systematic literature review (SLR) by offering a preliminary exploratory analysis, allowing the identification of trends and research gaps prior to applying more restrictive criteria in a more rigorous and exhaustive SLR ( Kitchenham et al., 2010).

To ensure methodological rigor, the study followed the guidelines of the PRISMA 2020 protocol (Preferred Reporting Items for Systematic Reviews and Meta-Analyses) ( Farisyi et al., 2022; Moher et al., 2015), ensuring validity and transparency in the selection and analysis of the reviewed studies. Additionally, the methodological guidelines proposed by Petersen et al. (2015) and Sinoara et al. (2017) were considered, as they establish specific criteria for identifying, organizing, and classifying the literature in systematic reviews.

Although PRISMA 2020 was originally developed for systematic literature reviews (SLRs), in this study it was adapted as a guiding framework for conducting a systematic literature mapping (SLM), due to its standardized structure and ability to ensure transparency in study selection. Additionally, methodological elements from Petersen et al. (2015) were incorporated; their mapping study guidelines are widely used in fields such as software engineering but are also applicable to educational research. Therefore, this study combines the methodological traceability of PRISMA with the classificatory logic of the Petersen model, enabling a rigorous and reproducible analysis of the scientific production.

### Eligibility criteria

To ensure the relevance and quality of the studies included, five inclusion criteria were established:

IC1. Scientific articles written in English or Spanish.

IC2. Studies published in Scopus, Web of Science, and SciELO in their final version.

IC3. Articles published between January 2005 and December 2024.

IC4. Studies available in Open Access format.

IC5. Research related to the integration of technology in the teaching of intercultural competences.

Likewise, exclusion criteria were defined to eliminate studies that did not meet the methodological standards of systematic literature mapping. The following were excluded:

EC1. Duplicate articles in the consulted databases.

EC2. Theses, books, book chapters, and conference papers, as they do not comply with the peer-review process of indexed scientific journals.

EC3. Articles not available in open access, whose full content could not be reviewed.

EC4. Studies outside the scope of interest, i.e., those that do not directly address the integration of technologies in the teaching of intercultural competences.

EC5. Publications in a preprint state or without peer review, which do not meet scientific validity criteria.

The studies were grouped for synthesis based on their alignment with the research questions (RQ1–RQ6), prioritizing those that explicitly addressed the integration of digital technologies in the teaching of intercultural competences in higher education contexts. Studies that, although mentioning any of the variables of interest, did not directly answer the proposed questions or did not meet the established methodological criteria were excluded.

### Information sources

The search for studies was conducted in three academic databases recognized for their high impact and scientific rigor: Scopus, Web of Science (WoS), and SciELO. These sources were selected due to their coverage of peer-reviewed literature, their relevance in the educational field, and their geographical and linguistic diversity. The last search in each database was performed on January 15, 2025, ensuring the currency of the retrieved records.

### Search strategy

The search strategy was designed based on a preliminary review of the literature and consultation of specialized thesauri, such as ERIC and UNESCO, to identify key terms and relevant synonyms for the object of study. Combinations of keywords related to intercultural competences, digital technologies, digital learning, higher education, and intercultural teaching were used, employing Boolean operators (AND, OR) to optimize the retrieval of relevant documents. The searches were adapted to the syntax of each database to ensure reproducibility. See Table 1.

**Table 1.** Search strategy used in database.

Database	Query performed	Search equation
Scopus, Web of Science (WoS) y SciELO	Title, abstract and keywords	(“intercultural competence” OR “intercultural skills”) AND (“digital technology” OR “digital learning”) AND (“higher education” OR “university”)

### Selection and data extraction process

**Figure 1.**
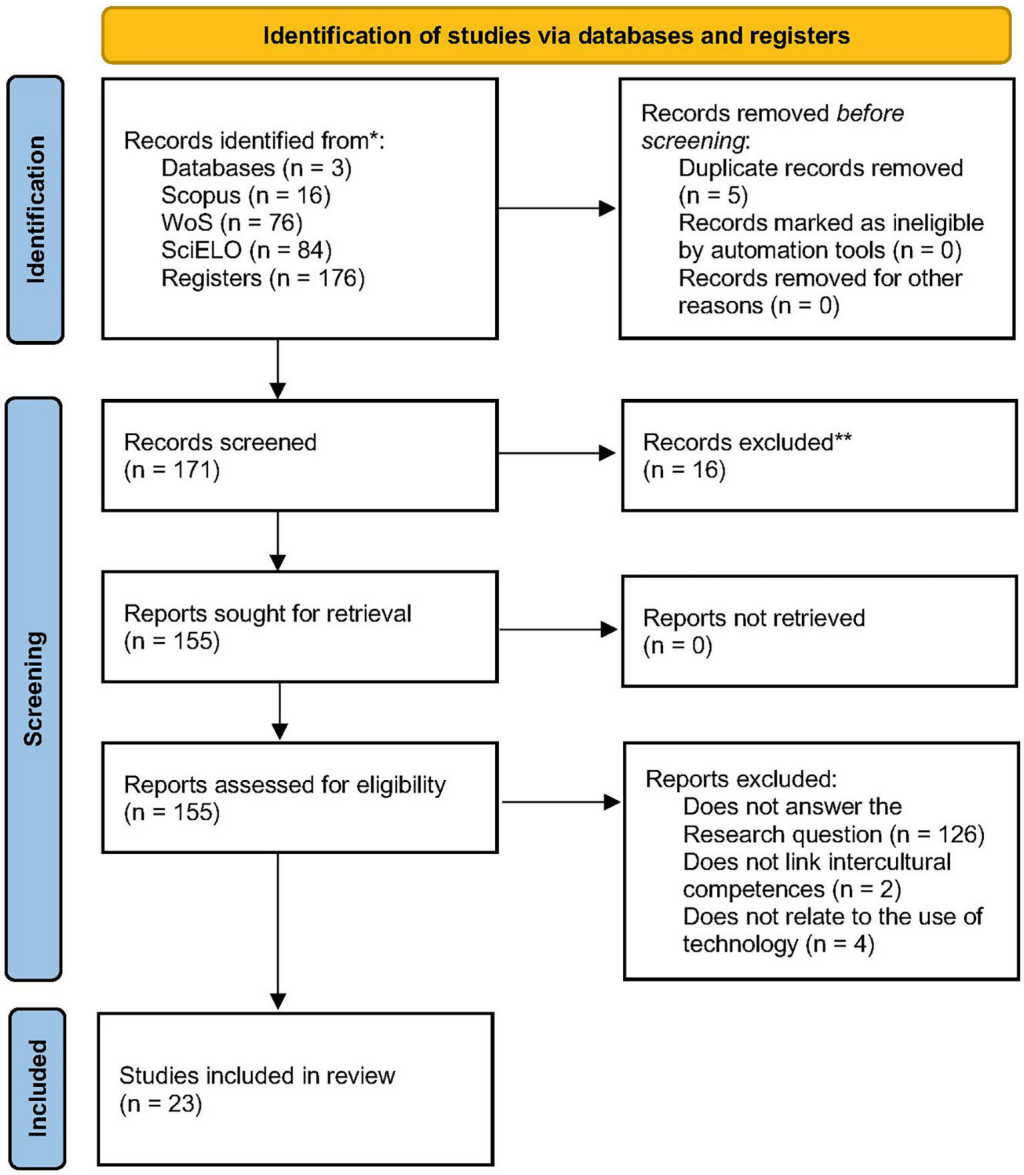
Flowchart of the literature selection process according to PRISMA 2020. **Source**: Page et al. (2021).

All documents retrieved from the Scopus, Web of Science (WoS), and SciELO databases were organized and managed using Microsoft Excel 2019. The selection of primary studies followed the three phases established in the PRISMA 2020 flow diagram ( Page et al., 2021): identification, screening, and inclusion (see Figure 1).

During the identification phase, a total of 176 records were retrieved from the three selected databases: 16 from Scopus, 76 from Web of Science, and 84 from SciELO. Prior to the screening phase, five duplicate records were removed, resulting in 171 unique studies eligible for title, abstract, and keyword screening.

In the screening phase, three reviewers independently analyzed the 171 records. Based on the inclusion criteria, 16 studies were excluded at this stage. The main reasons included lack of relevance to the research questions or insufficient linkage to intercultural competence. Discrepancies in inclusion were discussed and resolved by consensus among the reviewers. As a result, 155 studies progressed to the full-text eligibility assessment.

In the eligibility phase, the full texts of the remaining studies were reviewed by five reviewers. This review was also conducted independently. Discrepancies were documented and subsequently discussed until consensus was reached. No automated tools were used at this stage. The methodological quality assessment was carried out using a rubric with ten criteria based on Kitchenham et al. (2009), employing a Likert-type scale: yes (1 point), partial (0.5 points), and no (0 points). A minimum inclusion threshold of 7 out of 10 points was established to ensure the quality and relevance of the included studies.

The criteria evaluated included:

Q1: Clarity of objectives regarding the integration of digital technologies.

Q2: Methodological coherence and transparency.

Q3: Inclusion of higher education teachers or students.

Q4: Clear identification of methodological design.

Q5: Adequacy in data collection and analysis.

Q6: Clarity in the presentation of results.

Q7: Explicit reference to the use of digital technologies in education.

Q8: Comparison with traditional pedagogical methodologies.

Q9: Relevance to the teaching of intercultural competences.

Q10: Consistency between objectives, results, and conclusions.

Following this assessment, 132 studies were excluded: 126 for not addressing the research questions, 2 for not dealing with intercultural competences, and 4 for not reporting the use of digital technologies. Ultimately, 23 studies were included for detailed analysis. See Table 2.

**Table 2.** Studies included for systematic review.

N°	Author’s	Title article	Year
1	Gómez, M.	Competencias interculturales en instructores comunitarios que brindan servicio a la población indígena del estado de Chiapas	2010
2	McCloskey, E.	Global Teachers: A Model for Building Teachers’ Intercultural Competence Online	2012
3	Ciftci, E., & Savas, P.	The role of telecollaboration in language and intercultural learning: A synthesis of studies published between 2010 and 2015	2018
4	Carmona, M., Cruz, V., & García, L.	Desarrollo de competencias sociolingüísticas e interculturales en ELE: propuesta didáctica con blended Learning	2019
5	Dugartsyrenova, V., & Sardegna V.	Raising intercultural awareness through voice-based telecollaboration: perceptions, uses, and recommendations	2019
6	Zakharova, I., Kobicheva, A., & Rozova, N.	Results Analysis of Russian Students’ Participation in the Online International Educational Project X-Culture	2019
7	Dai, Y.	Blended Learning for Intercultural Competence: A Case Study in Engineering Education	2021
8	Rauer, J., Kroiss, M., Kryvinska, N., Engelhardt-Nowitzki, C., & Aburaia M.	Cross-university virtual teamwork as a means of internationalization at home	2021
9	Sarmiento, S., García, K., & Pozo, O.	Implementación de la metodología Lesson Study en el centro de apoyo San Vicente de Ecuador	2021
10	Villasol, M.	Rendimiento académico y patrones de uso del campus virtual: Un estudio de caso controlado	2021
11	Borger, J.	Getting to the CoRe of Collaborative Online International Learning (COIL)	2022
12	Jorgensen, M., Mason, A., Pedersen, R., & Harrison, R.	The Transformative Learning Potential in the Hybrid Space Between Technology and Intercultural Encounters	2022
13	Leiva, J., del Olmo, M., Aguilera, F., & Villalba, M.	Promotion of Intercultural Competencies and Use of ICT: Towards a Digitally Inclusive University; [Promoción de Competencias Interculturales y Uso de las TIC: Hacia una Universidad Inclusiva]	2022
14	Munoz-Escalona, P., De Crespo, Z., Marin, M., & Dunn, M.	Collaborative online international learning: A way to develop students’ engineering capabilities and awareness to become global citizens	2022
15	Woicolesco, V., Cassol-Silva, C., & Morosini, M.	Internationalization at Home and Virtual: A Sustainable Model for Brazilian Higher Education	2022
16	Guillen-Yparrea, N., & Ramirez-Montoya, M.	Intercultural Competencies in Higher Education:a systematic review from 2016 to 2021	2023a
17	Guillén-Yparrea, N; Ramírez-Montoya, M.	A review of collaboration through intercultural competencies in higher Education	2023b
18	Louahala, N.	Developing Learners’ Intercultural Communicative Competence through Online Exchanges: Case of Third-Year Students in Algeria	2023
19	Rubtsova, A., Zheleznyakova, O., Anosova, N., & Dashkina, A.	Collaborative Learning in Teaching Culture Studies to Further Training Program Students	2023
20	Simoes, A., & Sangiamchit, C.	Internationalization at Home: Enhancing Global Competencies in the EFL Classroom through International Online Collaboration	2023
21	Wiesner-Luna, V., & Burgoa-Godoy, C.	International collaborative learning experience between Higher Education Institutions in Colombia and Chile	2023
22	Kosman, B., de Jong, D., Knight-Agarwal, C., Chipchase, LS., Etxebarria, N.	The benefits of virtual learning abroad programs for higher education students: A phenomenological research study	2024
23	Savelyeva, N., & Sazonova, N.	Contemporary State of the Phenomenon "Digital Intercultural Competence" in Pedagogical Science	2024

For data extraction, a structured matrix was designed with the following variables: country or region of publication, type of technology used, area of focus (communication skills, cultural attitudes, or technological adoption), reported limitations, future lines of research, and type of intercultural competence addressed. Each study was assessed based on its alignment with research questions RQ1–RQ6. The absence of explicit information was coded as “not reported.” Data extraction was conducted independently by five reviewers and subsequently consolidated. Discrepancies were resolved through discussion among the reviewers.

The data were organized into frequency tables without applying inferential statistical analyses. No heterogeneity or sensitivity analyses were performed, given that the nature of this systematic mapping did not include quantitative comparisons between groups. It is important to note that during data extraction, both the competences and focus areas explicitly stated in the objectives or methodological designs of the studies, as well as those that emerged as observed outcomes or later reflections, were recorded. This distinction was maintained in the analysis matrices, although not all studies clearly differentiated between these levels.

Similarly, the categorization of both intercultural competence dimensions and digital tools was non-exclusive. A single study could be associated with multiple competences and tool types. For instance, some technologies, such as COIL, were classified under more than one category due to their multifunctional use across various pedagogical purposes. This approach was methodologically intentional and aimed to more accurately reflect the versatility of digital tools in educational practice. As a result, some of the reported percentages may exceed 100%.

## Results

The most relevant findings from the included studies are presented below, organized according to the formulated research questions, with the aim of facilitating a structured and coherent interpretation of the body of evidence analyzed.

### RQ1. In which countries and/or regions is research concentrated?

The systematic review shows that research on intercultural competences mediated by digital technologies in higher education is primarily concentrated in the Americas and Europe, which together account for 78% of the total studies analyzed. At the national level, Mexico leads scientific production with a frequency of 15% (n=4), followed by the United States, Spain, and Russia, each with 11% (n=3). These figures indicate a clear orientation of the literature toward Western contexts, particularly in countries with active policies of educational internationalization. Australia contributes 7% (n=2), highlighting the growing involvement of Oceania in this field. The United Kingdom also accounts for 7% (n=2), reflecting continued European engagement in the development of digital intercultural competences. Meanwhile, countries such as the United Kingdom, Scotland, Turkey, Algeria, Portugal, Thailand, Chile, Colombia, Ecuador, Venezuela, Brazil, and Austria each present a frequency of 4% (n=1), reflecting emerging, albeit still limited, geographical diversity.

**Figure 2.**
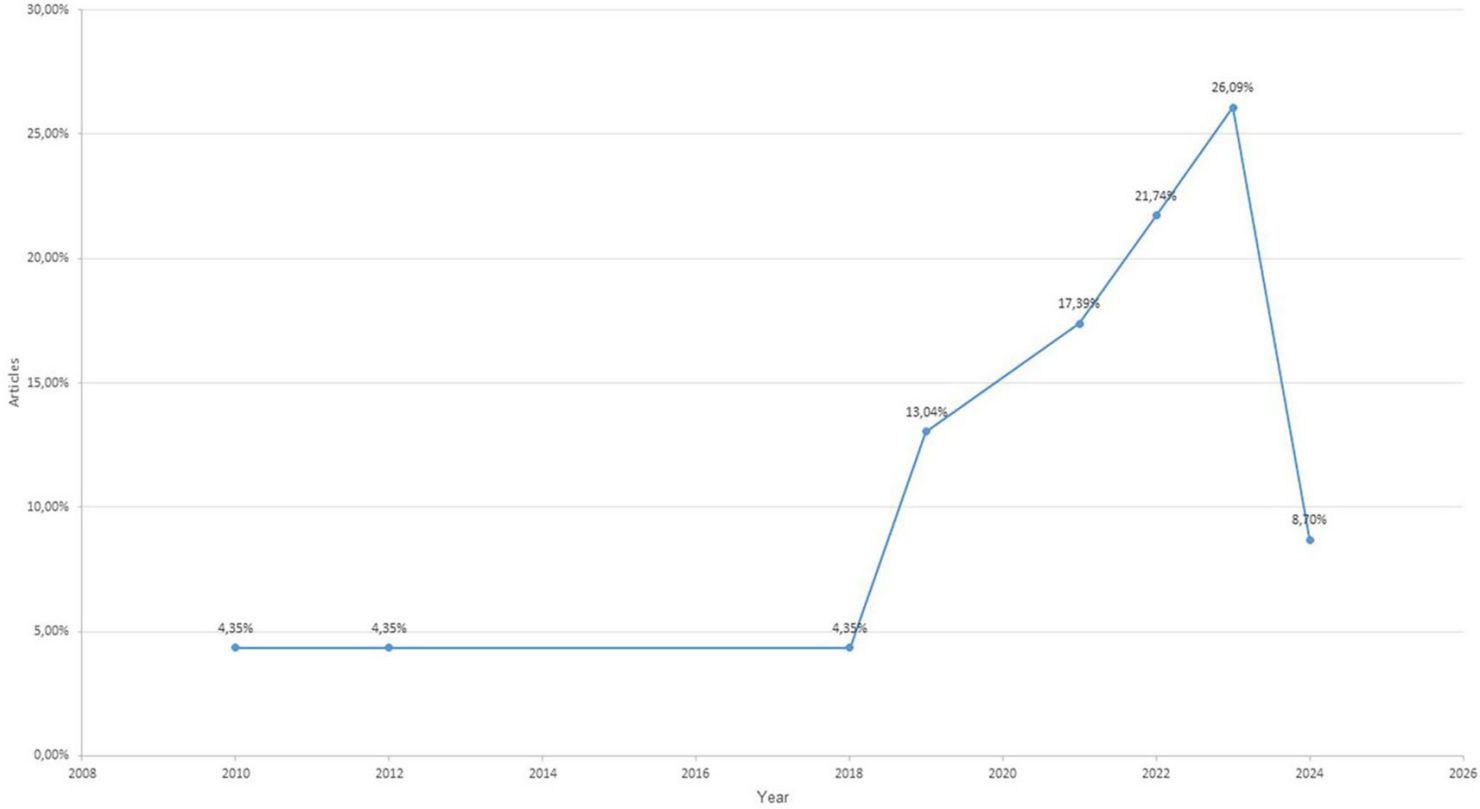
Temporal distribution of the studies included in the systematic review (2010-2024).

The temporal analysis revealed a significant increase in scientific production from 2019 onward, with 86.96% of the analyzed studies published in this period. The most productive years were 2023 (26.09%), 2022 (21.74%), and 2021 (17.39%), reflecting a growing interest in integrating digital technologies to develop intercultural competences, particularly in higher education settings. Earlier years showed minimal representation. This distribution appears to reflect a real academic trend rather than a limitation of the search strategy, as the databases were comprehensively queried for the entire 2005–2024 period (see Figure 2).

### RQ2. What types of digital technologies are used in the teaching of intercultural competences?

Of the 23 studies included in this systematic review, 22 (95.65%) explicitly report the incorporation of digital technologies as part of their strategies for developing intercultural competences in higher education. The sole exception is the study by Ciftci and Savas (2018), which, although acknowledging the benefits of technologies in intercultural teaching, does not specify the use of concrete digital tools. Its inclusion was retained due to the conceptual contribution it provides in understanding the role of technology in intercultural contexts. However, this lack of specificity limits its comparability with other studies and is considered a methodological limitation in interpreting the results.

Among the identified technological categories, digital platforms are the most represented, appearing in 15 studies (68.18%). This category encompasses learning management systems (LMS) such as Blackboard, Moodle, and Canvas, valued for their ability to centralize content, facilitate asynchronous communication, and structure collaborative tasks in virtual environments. Notable examples include the works of Jørgensen et al. (2020), Zakharova et al. (2019), and Kosman et al. (2024), which demonstrate how these platforms foster intercultural interaction in multicultural educational contexts.

This is followed by pedagogical technologies and methodological approaches such as COIL (Collaborative Online International Learning), blended learning, videoconferencing platforms (Zoom, Skype), virtual environments, and simulators, reported in 10 studies (45.45%). These tools enable the creation of structured and intentional learning experiences for intercultural interaction, as evidenced by the research of Borger (2022), Dai (2021), and Guillén-Yparrea & Ramírez-Montoya (2023a, 2023b).

Digital educational resources, present in 6 studies (27.27%), include gamification, interactive materials, wikis, explanatory videos, Microsoft Forms, and open repositories. These resources serve as didactic complements that allow for the adaptation of content to the cultural particularities of students, as highlighted by Leiva et al. (2022) and Sarmiento et al. (2021).

The category of social networks, also reported in 6 studies (27.27%), involves the use of platforms such as WhatsApp or discussion forums as spaces for informal interaction among students from different cultures. Although still underexplored in formal contexts, studies such as those by Rauer et al. (2021) and Louahala (2023) emphasize their value in fostering spontaneous and authentic cultural exchange.

Finally, productivity tools such as Google Drive, Trello, and OneNote appear in 4 studies (18.18%). Although their potential for asynchronous collaboration is recognized, their implementation in intercultural educational contexts remains limited, possibly due to gaps in teacher training regarding their pedagogical use, as suggested by Munoz-Escalona et al. (2020) and Louahala (2023).

These findings reflect a clear preference for technologies focused on structured teaching, with still an incipient exploration of tools oriented toward social collaboration and cultural personalization of learning. Table 3 presents a detailed synthesis of the authors, categories, number of studies, and usage percentages.

**Table 3.** Digital technologies used in the teaching of intercultural competences.

Authors	Technological category	Number of studies	Percentage
McCloskey (2012); Dugartsyrenova & Sardegna (2019); Zakharova et al. (2019); Dai (2021); Rauer et al. (2021); Villasol (2021); Leiva et al. (2022); Jorgensen et al. (2022); Munoz-Escalona et al. (2020); Rubtsova et al. (2023); Simoes & Sangiamchit (2023); Guillen-Yparrea, & Ramirez-Montoya (2023a); Guillen-Yparrea, & Ramirez-Montoya (2023b); Kosman et al. (2024); Savelyeva & Sazonova (2024)	Digital platforms	15	68.18%
Gómez (2010); Woicolesco et al. (2022); Carmona et al. (2019); Dugartsyrenova & Sardegna (2019); Dai (2021); Borger (2022); Wiesner-Luna & Burgoa-Godoy (2023); Guillen-Yparrea, & Ramirez-Montoya (2023a); Guillen-Yparrea, & Ramirez-Montoya (2023b); Rubtsova et al. (2023)	Pedagogical technologies and approaches	10	45.45%
Rauer et al. (2021); Leiva et al. (2022); Jorgensen et al. (2022); Wiesner-Luna & Burgoa-Godoy (2023); Carmona et al. (2019); Sarmiento et al. (2021)	Educational resources	6	27.27%
Zakharova et al. (2019); Leiva et al. (2022); Louahala (2023); Munoz-Escalona et al. (2020); Guillen-Yparrea & Ramírez-Montoya (2023b); Rauer et al. (2021)	Social media	6	27.27%
Woicolesco et al. (2022); Munoz-Escalona et al. (2020); Louahala (2023); Wiesner-Luna & Burgoa-Godoy (2023)	Productivity tools	4	18.18%
Ciftci & Savas (2018)	Not mentioned in the study		

Note: Percentages exceed 100% because studies often address more than one category.

### RQ3. In which areas does research on the integration of digital technologies in the teaching of intercultural competences focus, such as communication skills, cultural attitudes, and technological adoption?

The analysis of the 23 studies included in this systematic review reveals a broad diversity of focus areas regarding the use of digital technologies to foster intercultural competences in higher education. However, there is a clear predominance of research centered on the development of communication skills, with a total of 14 studies (60,87%). This category includes works such as those by Borger (2022), Wiesner-Luna & Burgoa-Godoy (2023), and Kosman et al. (2024), which highlight the importance of virtual environments in strengthening intercultural interaction among students from different cultural contexts. For their part, Guillén-Yparrea and Ramírez-Montoya (2023a) emphasize that language teaching, international experiences, and collaborative virtual environments are the most common contexts for fostering such communication skills in higher education.

In second place, technological adoption accounts for 21,74% of the studies (5 publications). This focus is linked to the strengthening of intercultural competences through both instrumental and critical mastery of ICT, including research such as that by Jørgensen et al. (2020) and Savelyeva & Sazonova (2024), who explore the integration and critical appropriation of digital tools in intercultural teaching-learning processes. Sociolinguistic and intercultural competences are addressed in 3 studies (13,04%), highlighting the need to understand linguistic and cultural codes in multilingual digital environments ( Leiva et al., 2022; Guillen-Yparrea & Ramírez-Montoya, 2023a).

Other less frequent areas include teacher training (8,70%, 2 studies), curriculum internationalization (8,70%, 2 studies), and the development of cultural attitudes (8,70%, 2 studies), each reflecting specific lines of research focused on pedagogical design, cultural awareness, or the construction of global educational programs. Finally, categories such as critical thinking, pedagogy, learner autonomy, professional development in healthcare, and technical text translation each appear in only one study (4,35%), suggesting future opportunities to expand research in these less explored domains.

Table 4 summarizes these results, highlighting the authors, thematic area addressed, study frequency, and their percentage representation within the analyzed corpus.

**Table 4.** Research approaches to the integration of digital technologies in the teaching of intercultural competences.

Authors	Focus area	Frequency	Percentage
Borger (2022); Wiesner-Luna & Burgoa-Godoy (2023); Carmona et al. (2019); McCloskey (2012); Munoz-Escalona et al. (2020); Jorgensen et al. (2022); Simoes & Sangiamchit (2023); Zakharova et al. (2019); Ciftci & Savas (2018); Dugartsyrenova & Sardegna (2019); Guillen-Yparrea & Ramirez-Montoya (2023a); Kosman et al. (2024); Louahala (2023); Rauer et al. (2021)	Communication skills	14	60,87%
Jorgensen et al. (2022); Munoz-Escalona et al. (2020); Dugartsyrenova & Sardegna (2019); Guillen-Yparrea & Ramirez-Montoya (2023b); Savelyeva & Sazonova (2024)	Technology adoption	5	21,74%
Leiva et al. (2022); Munoz-Escalona et al. (2020); Guillen-Yparrea & Ramirez-Montoya (2023a)	Sociolinguistic and intercultural competences	3	13,04%
Sarmiento et al. (2021); Gómez (2010)	Teacher training	2	8,70%
Dugartsyrenova & Sardegna (2019); Guillen-Yparrea & Ramirez-Montoya (2023a)	Cultural attitudes	2	8,70%
Rauer et al. (2021); Woicolesco et al. (2022)	Internationalization of the curriculum	2	8,70%
Jorgensen et al. (2022)	Critical thinking	1	4,35%
Villasol (2021)	Pedagogy	1	4,35%
Kosman et al. (2024)	Professional development in healthcare	1	4,35%
Louahala (2023)	Autonomous learning in virtual environments	1	4,35%
Rubtsova et al. (2023)	Technical text translation and editing	1	4,35%

Note: Percentages exceed 100% because studies often address more than one category.

It is important to mention that in several studies, the intercultural competences and areas of digital integration were identified as emerging benefits during the implementation of the interventions rather than as predefined pedagogical goals. This methodological ambiguity may affect the interpretation of the results and highlights the need for future studies with more explicit designs and structured pre-post evaluations.

### RQ4. Which intercultural competences are addressed?

The results show that research on intercultural competences mediated by digital technologies in higher education is primarily concentrated in five dimensions: adaptation and management (47.83%), intercultural knowledge (39.13%), communication skills (39.13%), intercultural attitudes (39.13%), and collaborative work (21.74%). To a lesser extent, competences were identified in the areas of personal development (8.70%) and innovative leadership (4.35%), indicating emerging areas in the literature (see Table 5).

**Table 5.** Intercultural competences addressed by the studies analysed.

Dimension	Percentage	Competence	Authors	Construct according to authors
Adaptation and management	47.83%	Cultural adaptability	Dai (2021); Dugartsyrenova & Sardegna (2019); Jorgensen et al. (2022); Kosman et al. (2024); Savelyeva & Sazonova (2024); Simoes & Sangiamchit (2023); Woicolesco et al. (2022)	Ability to flexibly modify behaviors and strategies when interacting with people from different cultures, while maintaining effectiveness in achieving objectives
		Adaptation to diverse contexts	Rauer et al. (2021); Rubtsova et al. (2023)	Ability to recognize and adapt to the implicit and explicit norms of different cultural environments, especially in professional settings
		Intercultural digital skills	Savelyeva & Sazonova (2024); Sarmiento et al. (2021)	Competence to effectively use communication technologies in multicultural virtual environments, overcoming both cultural and technological barriers
Intercultural knowledge	39.13%	Intercultural awareness	Borger (2022); Dugartsyrenova & Sardegna (2019); Guillen-Yparrea & Ramirez-Montoya (2023a); McCloskey (2012); Savelyeva & Sazonova (2024)	Understanding of one’s own and others’ cultural values, norms, and practices, recognizing their influence on perceptions and behaviors
		Cultural awareness	Rubtsova et al. (2023); Kosman et al. (2024)	Ability to identify and compare key cultural elements (e.g., individualistic vs. collectivist values) and reflect on their impact on interactions
		Global awareness	Munoz-Escalona et al. (2020); McCloskey (2012)	Understanding global issues and their relationship with intercultural dynamics, considering historical and sociopolitical perspectives
Communication skills	39.13%	Intercultural communication	Ciftci & Savas (2018); Zakharova et al. (2019); Dai (2021); Guillen-Yparrea & Ramirez-Montoya (2023a); Rauer et al. (2021); Savelyeva & Sazonova (2024); Wiesner-Luna & Burgoa-Godoy (2023); Woicolesco et al. (2022)	Interactive process where people from different cultures exchange verbal and non-verbal messages, negotiating meanings with awareness of cultural differences
		Effective communication in intercultural contexts	Zakharova et al. (2019)	Ability to appropriately interact in multicultural environments by combining cultural knowledge, adaptability skills, and respectful attitude
Intercultural attitudes	39.13%	Intercultural sensitivity	Ciftci & Savas (2018); Dai (2021); Leiva et al. (2022); McCloskey (2012); Munoz-Escalona et al. (2020)	Willingness to recognize, respect, and value cultural practices different from one’s own
		Empathy	Leiva et al. (2022); Simoes & Sangiamchit (2023); Wiesner-Luna & Burgoa-Godoy (2023)	Ability to understand and share the feelings of people from other cultures, especially in situations of conflict or intercultural stress
		Inclusion and welcoming	Leiva et al. (2022)	Proactive attitude towards creating environments that foster the equitable participation of diverse individuals
Collaborative work	21,74%	Global teamwork	Munoz-Escalona et al. (2020); Simoes & Sangiamchit (2023); Wiesner-Luna & Burgoa-Godoy (2023)	Ability to coordinate efforts with geographically distributed multicultural teams, managing challenges such as time zones, language barriers, and diversity in work styles, communication, and decision-making
		Collaborative work	Kosman et al. (2024); Savelyeva & Sazonova (2024)	Skill to co-create knowledge and solutions with peers from different cultures, integrating diverse perspectives through intercultural dialogue, meaning negotiation, and consensual creation processes
Personal development	8,70%	Reflection on identity	Jorgensen et al. (2022)	Critical self-awareness process that enables analyzing how one’s own cultural identity influences perceptions, prejudices, and ways of interacting in multicultural contexts
		Autonomy in learning	Louahala (2023)	Competence to actively manage one’s intercultural learning through selective resource seeking, comparative culture analysis, and continuous self-assessment of intercultural development
Innovative leadership	4,35%	Leadership and creativity	Zakharova et al. (2019)	Ability to design and implement innovative strategies that foster inclusion and high performance in multicultural teams, resolving intercultural conflicts through creative solutions

The most recurrent dimension is adaptation and management, with cultural adaptability emerging as the most frequently addressed competence (n = 7), defined as the ability to flexibly adjust strategies and behaviors in multicultural contexts ( Dai, 2021; Dugartsyrenova & Sardegna, 2019; Jorgensen et al., 2022; Kosman et al., 2024; Savelyeva & Sazonova, 2024; Simoes & Sangiamchit, 2023; Woicolesco et al., 2022). Other relevant competences within this dimension include adaptation to diverse contexts (n = 2) and intercultural digital skills (n = 2), highlighting the growing need for technological competences to operate effectively in global and interconnected environments. Studies such as those by Sarmiento et al. (2021) underscore the role of digital tools in overcoming cultural and technological barriers, especially in virtual educational settings.

Regarding intercultural knowledge, competences associated with intercultural awareness (n = 5), cultural awareness (n = 2), and global awareness (n = 2) were identified. These competences involve recognizing one’s own and others’ cultural values, norms, and practices, as well as understanding global issues from historical and sociopolitical perspectives ( Borger, 2022; McCloskey, 2012; Munoz-Escalona et al., 2020). Rubtsova et al. (2023) highlight the use of immersive technologies as a means of contrasting cultural and sociopolitical perspectives, thereby expanding critical understanding of cultural diversity.

The dimension of communication skills was addressed in 39.13% of the studies, mainly through intercultural communication (n = 8), understood as a dynamic process of negotiating meanings between individuals from different cultures ( Ciftci & Savas, 2018; Zakharova et al., 2019; Dai, 2021; Guillen-Yparrea & Ramírez-Montoya, 2023a; Rauer et al., 2021; Savelyeva & Sazonova, 2024; Wiesner-Luna & Burgoa-Godoy, 2023; Woicolesco et al., 2022). Notable is the evidence of innovative methodologies such as COIL (Collaborative Online International Learning), which promote the construction of shared meanings in international academic environments ( Dai, 2021; Guillén-Yparrea & Ramírez-Montoya, 2023a).

With respect to intercultural attitudes, competences such as intercultural sensitivity (n = 5), empathy (n = 3), and inclusion and hospitality (n = 1) are emphasized. These competences reflect a positive disposition toward recognizing and valuing cultural diversity, which is crucial for inclusive educational environments ( Ciftci & Savas, 2018; Leiva et al., 2022; McCloskey, 2012; Munoz-Escalona et al., 2020). Recent works, such as that by Wiesner-Luna and Burgoa-Godoy (2023), illustrate the potential of immersive technologies such as virtual reality to generate empathic experiences in the face of discrimination, demonstrating a transformative approach to intercultural training.

The dimension of collaborative work emerged linked to competences such as global teamwork (n = 3) and intercultural collaboration (n = 2). These competences emphasize the ability to manage projects and build knowledge in diverse and geographically distributed environments, overcoming barriers such as differences in time zones, languages, or methodologies ( Kosman et al., 2024; Wiesner-Luna & Burgoa-Godoy, 2023; Simoes & Sangiamchit, 2023). This highlights the key role of digital platforms in facilitating intercultural coordination in virtual spaces.

Finally, although less represented, dimensions such as personal development were identified, including reflection on cultural identity (n = 1) and autonomy in intercultural learning (n = 1), as well as innovative leadership, with competences related to creative leadership (n = 1). Jorgensen et al. (2022) and Louahala (2023) highlight how reflective practices mediated by digital technologies strengthen cultural self-awareness, while Zakharova et al. (2019) explores the role of inclusive and creative leadership in hybrid educational contexts.

The results reflect a growing field of research in which digital technologies are emerging as key tools for developing intercultural competences. Nonetheless, there remains uneven attention among the various dimensions, and opportunities are identified to deepen research in less explored areas, particularly in leadership and personal development, as well as in more diverse cultural contexts.

### RQ5. What are the limitations of the research?

Of the 23 studies analyzed, 21 (91.3%) explicitly reported limitations in their research, while 2 (8.7%) did not mention any. To facilitate a clearer understanding of the results, the limitations were organized into six categories: methodological, sampling bias, technological barriers, communication barriers, institutional collaborative challenges, and pedagogical limitations (see Table 6). These categories reveal recurring obstacles that affect the validity, applicability, and generalization of findings related to intercultural competences mediated by digital technologies.

**Table 6.** Limitations of the research analysed.

Category	Main limitations	Authors	Frequency	Percentage	Percentage by category
Institutional collaborative challenges	Difficulty finding partners	Simoes & Sangiamchit (2023)	1	4.35%	34.78%
	Lack of institutional response	Woicolesco et al. (2022)	1	4.35%	
	Lack of commitment from some team members	Munoz-Escalona et al. (2020); Wiesner-Luna & Burgoa-Godoy (2023); Simoes & Sangiamchit (2023)	3	13.04%	
	Insufficient local cultural information	McCloskey (2012)	1	4.35%	
	Insufficient time	Carmona et al. (2019); Wiesner-Luna & Burgoa-Godoy (2023)	2	8.70%	
Methodological	Small sample size	Leiva et al. (2022); Jorgensen et al. (2022); Guillén-Yparrea & Ramírez-Montoya (2023b)	3	13.04%	30.43%
	Lack of sample diversity	McCloskey (2012)	1	4.35%	
	Qualitative approach without triangulation	McCloskey (2012)	1	4.35%	
	Lack of quantitative assessment (non-validated instruments)	Woicolesco et al. (2022); Simoes & Sangiamchit (2023)	2	8.70%	
Sampling bias	Lack of cultural diversity	Villasol (2021); Guillén-Yparrea & Ramírez-Montoya (2023a)	2	8.70%	26.09%
	Exclusion of students	Leiva et al. (2022); Munoz-Escalona et al. (2020); Simoes & Sangiamchit (2023)	3	13.04%	
	Inconsistent participation	Zakharova et al. (2019)	1	4.35%	
Pedagogical	Lack of teacher training	Gómez (2010); Sarmiento et al. (2021)	2	8.70%	21.74%
	Lack of time for module development	Wiesner-Luna & Burgoa-Godoy (2023)	1	4.35%	
	Pedagogical and logistical challenges	McCloskey (2012)	1	4.35%	
	Teacher ethnocentrism	McCloskey (2012)	1	4.35%	
Technological barriers	Problems accessing platforms	Munoz-Escalona et al. (2020); Louahala (2023); Simoes & Sangiamchit (2023)	3	13.04%	17.39%
	Digital divide	Sarmiento et al. (2021)	1	4.35%	
Communication barriers	Language barriers	Zakharova et al. (2019); Simoes & Sangiamchit (2023)	2	8.70%	13.04%
	Difficulties in initial communication due to lack of early trust	Wiesner-Luna & Burgoa-Godoy (2023)	1	4.35%	

The most frequent category was institutional collaborative challenges (34.78%), encompassing limitations such as difficulty in finding institutional partners ( Simoes & Sangiamchit, 2023), lack of response from institutions ( Woicolesco et al., 2022), low engagement from some members of academic teams ( Munoz-Escalona et al., 2020; Simoes & Sangiamchit, 2023; Wiesner-Luna & Burgoa-Godoy 2023), insufficient local cultural information ( McCloskey, 2012), and lack of time to properly implement the modules ( Carmona et al., 2019; Wiesner-Luna & Burgoa-Godoy, 2023).

Secondly, methodological limitations (30.43%) included aspects such as small sample sizes ( Guillén-Yparrea & Ramírez-Montoya, 2023b; Jørgensen et al., 2020; Leiva et al., 2022), qualitative approaches without data triangulation ( McCloskey, 2012), lack of quantitative evaluation using validated instruments ( Simoes & Sangiamchit, 2023; Woicolesco et al., 2022), and low diversity in samples ( McCloskey, 2012). These constraints compromise the robustness of methodological designs and the ability to generalize findings.

Sampling bias accounted for 26.09% of the limitations, focused on the exclusion of students from research processes ( Leiva et al., 2022; Munoz-Escalona et al., 2020; Simoes & Sangiamchit, 2023), inconsistent participation of subjects ( Zakharova et al., 2019), and the lack of cultural diversity among participants ( Guillén-Yparrea & Ramírez-Montoya, 2023a; Villasol, 2021). These limitations affect the representativeness and depth of intercultural analysis.

Pedagogical limitations (21.74%) include lack of teacher training in digital tools ( Gómez, 2010; Sarmiento et al., 2021), logistical difficulties in designing and implementing intercultural modules ( Wiesner-Luna & Burgoa-Godoy, 2023), pedagogical challenges in sustaining intercultural interactions ( McCloskey, 2012), and ethnocentric bias detected in some teachers ( McCloskey, 2012), which can hinder the construction of meaningful telecollaboration experiences.

Technological barriers (17.39%) primarily involved difficulties accessing digital platforms ( Louahala, 2023; Munoz-Escalona et al., 2020; Simoes & Sangiamchit, 2023) and the existence of a significant digital divide in certain contexts ( Sarmiento et al., 2021), which limits equity in access and participation. Finally, communication barriers (13.04%) reflect challenges such as language barriers ( Simoes & Sangiamchit, 2023; Zakharova et al., 2019) and a lack of initial trust among students that hinders smooth interaction ( Wiesner-Luna & Burgoa-Godoy, 2023). These limitations negatively impact the quality of intercultural exchanges in digital environments.

### RQ6. What are the future lines of research on intercultural competences?

Of the 23 studies reviewed, 21 (91.3%) explicitly identify future lines of research related to the development of intercultural competences mediated by technology (see Table 7). These findings reflect a consensus within the scientific community regarding the need to continue innovating, evaluating, and contextualizing these competences in diverse educational scenarios. For analytical clarity, the proposals were organized into six thematic categories according to their frequency and relative incidence

**Table 7.** Future lines of research on intercultural competences.

Category	Percentage by category	Reseach lines	Key authors	Frequency	Percentage by frequency
Assessment and metrics	82.61%	Design of instruments to measure intercultural critical thinking	Simoes & Sangiamchit (2023); Rauer et al. (2021)	2	8.70%
		Assess the impact of internationalization at home and interculturality in virtual environments	Woicolesco et al. (2022); Guillén-Yparrea & Ramirez-Montoya (2023a)	2	8.70%
		Evaluate the effectiveness of digital pedagogical strategies in multicultural contexts	Gómez (2010); Rauer et al. (2021); Munoz-Escalona et al. (2020); Guillén-Yparrea, & Ramirez-Montoya (2023b); Zakharova et al. (2019)	5	21.74%
		Analyze how digital intercultural experiences affect cultural identity perception	Rauer et al. (2021)	1	4.35%
		Explore the sustainability and impact of technology-mediated intercultural competence training programs in higher education	Rauer et al. (2021); Villasol (2021); Savelyeva & Sazonova (2024)	3	13.04%
		Compare presential and virtual experiences in intercultural competence development	Kosman et al. (2024); Kosman et al. (2024)	2	8.70%
		Evaluate intercultural competences through immersion and social interaction	Carmona et al. (2019)	1	4.35%
		Longitudinal studies on employment impact	Dugartsyrenova & Sardegna (2019); Dai (2021); Kosman et al. (2024)	3	13.04%
Technopedagogical innovation	60.87%	Integration of AI/gamification in intercultural environments	Dugartsyrenova & Sardegna (2019); Rubtsova et al. (2023); Savelyeva & Sazonova (2024)	3	13.04%
		Development of immersive (virtual/augmented reality) and/or innovative environments	Jørgensen et al. (2020); Guillén-Yparrea & Ramírez-Montoya (2023a); Savelyeva & Sazonova (2024); Ciftci & Savas (2018); Villasol (2021)	5	21.74%
		Development of effective strategies to overcome logistical and ethnocentric barriers in intercultural interactions	McCloskey (2012); Sarmiento et al. (2021)	2	8.70%
		Personalization of intercultural learning in hybrid or telecollaborative settings	Dai (2021); Louahala (2023); Ciftci & Savas (2018); Guillén-Yparrea & Ramirez-Montoya (2023a)	4	17.39%
Teacher training	30.43%	Development of multicultural PCK (Pedagogical Content Knowledge)	Borger (2022); Villasol (2021)	2	8.70%
		Development of programs to strengthen teacher training in intercultural and digital competences	Gómez (2010); Louahala (2023); Kosman et al. (2024); Leiva et al. (2022)	4	17.39%
		Training in COIL methodologies and telecollaboration	Wiesner-Luna & Burgoa-Godoy (2023)	1	4.35%
Specific contexts	13.04%	Application in professional fields (healthcare, business)	Guillén-Yparrea & Ramírez-Montoya (2023a)	1	4.35%
		Post-pandemic effects in higher education	Villasol (2021); Woicolesco et al. (2022)	2	8.70%
Institutional sustainability	8.70%	Models for inclusive internationalization	Woicolesco et al. (2022); McCloskey (2012)	2	8.70%
Digital equity	8.70%	Bridging technological gaps in regions with low connectivity	Gómez (2010); Dugartsyrenova & Sardegna (2019)	2	8.70%

Note: Categories are not mutually exclusive. Certain tools appear in more than one category due to their versatility in educational contexts.

The category with the greatest representation is Assessment and Metrics (82.61%), reflecting growing concern about improving measurement instruments and generating robust empirical evidence. Among the most notable proposals are the evaluation of the effectiveness of digital pedagogical strategies in multicultural contexts (21.74%) ( Gómez, 2010; Guillen-Yparrea & Ramírez-Montoya, 2023a; Munoz-Escalona et al., 2020; Rauer et al., 2021; Zakharova et al., 2019), followed by longitudinal studies on the labor impact of these competences (13.04%) ( Dai, 2021; Dugartsyrenova & Sardegna, 2019; Kosman et al., 2024) and studies promoting interest in exploring the sustainability and impact of technology-mediated intercultural competence training programs in higher education (13.04%) ( Rauer et al., 2021; Savelyeva & Sazonova, 2024; Villasol, 2021). There is also a proposal to develop specific instruments for measuring intercultural critical thinking and assessing its effect on perceptions of cultural identity or direct social interaction.

Secondly, Technopedagogical Innovation (60.87%) emerges as a priority area. Notable proposals include the development of immersive environments (21.74%) ( Ciftci & Savas, 2018; Guillén-Yparrea & Ramírez-Montoya, 2023a; Jørgensen et al., 2020; Savelyeva & Sazonova, 2024; Villasol, 2021) and personalized learning in hybrid and telecollaborative modalities (17.39%) ( Ciftci & Savas, 2018; Dai, 2021; Guillen-Yparrea & Ramírez-Montoya, 2023a; Louahala, 2023). These proposals point to the use of artificial intelligence, gamification, and innovative methodologies to enhance the intercultural experience in digital contexts.

Teacher Training (30.43%) is consolidated as a strategic line for institutional strengthening. It is recommended to design training programs that integrate digital and intercultural competences (17.39%) ( Gómez, 2010; Kosman et al., 2024; Leiva et al., 2022; Louahala, 2023), as well as to promote the development of pedagogical content knowledge (PCK) in multicultural contexts (8.70%) ( Villasol, 2021; Borger, 2022).

The categories of Specific Contexts and Institutional Sustainability appear in 13.04% of the studies. In the first case, applied research is suggested in professional areas such as healthcare and business ( Guillén-Yparrea & Ramírez-Montoya, 2023a) and studies on the impact of the pandemic on higher education ( Villasol, 2021; Woicolesco et al., 2022). In the second, it is proposed to develop models that ensure inclusive and sustainable internationalization, especially in institutions outside traditional academic cooperation frameworks ( McCloskey, 2012; Woicolesco et al., 2022).

Finally, Digital Equity accounts for 8.70% of the mentions, with proposals aimed at overcoming technological gaps in regions with low connectivity ( Dugartsyrenova & Sardegna, 2019; Gómez, 2010), highlighting the importance of ensuring equitable access to technology-mediated intercultural learning environments. These results demonstrate a dynamic field of study moving toward strengthening more inclusive, contextualized, and sustainable approaches for teaching intercultural competences in the digital age.

## Discussion

### RQ1. In which countries and/or regions is research concentrated?

This systematic review shows that research on intercultural competences in higher education is primarily concentrated in the Americas and Europe, with significant contributions from countries such as Mexico, the United States, and Spain. This pattern aligns with the findings of Dores et al. (2025), who report sustained growth in publications in these regions, driven by academic internationalization policies and the increasing demand for culturally competent professionals. In the European context, Milani and Portera (2021) highlight the institutional commitment to incorporating intercultural competence into teacher training, although they also point out methodological and pedagogical limitations for its effective implementation.

Beyond the geographic pattern, the temporal analysis of the included studies reinforces this trend. Most of the reviewed publications are concentrated between 2021 and 2023, indicating a recent surge in academic interest in this topic. This increase could be linked to the impact of the COVID-19 pandemic, which accelerated the implementation of international educational programs mediated by digital technologies and fostered reflection on the need for more interculturalized training ( Honen-Delmar & Rega, 2023; Rokos et al., 2023). The emergence of new modalities such as Collaborative Online International Learning (COIL) and virtual exchanges has served as a catalyst for this type of research, particularly in countries of the Global North.

On the other hand, although the presence of studies originating from Africa and Asia remains limited, significant experiences are emerging that broaden the geographical scope of the literature. A notable example is the study by Honen-Delmar and Rega (2023), who document successful higher education semi-presential initiatives in refugee camps in Kenya and Malawi, focused on developing intercultural competences. These emerging initiatives, though still in their early stages, deserve greater visibility and recognition, as they provide valuable perspectives from diverse cultural realities. The evidence suggests the need to geographically diversify research, promoting more inclusive approaches that reflect the plurality of educational contexts worldwide.

Regarding the temporal distribution, the exponential growth observed since 2019 (86.96% of studies) reflects the impact of the COVID-19 pandemic, which accelerated the adoption of digital technologies in higher education ( Basantes-Andrade et al., 2024a; Kulich et al., 2021). This phenomenon has driven the exploration of digital methodologies to foster intercultural competences and highlighted structural gaps that limit their global implementation ( Ayazbayeva et al., 2025). While this research field demonstrates dynamism and global relevance, its progress continues to be influenced by geopolitical inequalities and its connection to specific technological contexts.

Additionally, it is important to consider that the inclusion of studies exclusively in English or Spanish may have limited the geographical representativeness of the sample, favoring research originating from Latin America and Spanish-speaking countries. This linguistic bias may have influenced the predominance of Mexico and Spain in the results; therefore, future reviews could expand the language criterion to include other relevant languages, such as French, Chinese, or Arabic, to better reflect geocultural diversity.

### RQ2. What types of digital technologies are used in the teaching of intercultural competences?

The findings of this systematic review reveal a marked inclination toward the use of structured digital technologies in the development of intercultural competences in higher education, with a particular emphasis on learning management systems (LMS) such as Moodle, Blackboard, and Canvas. This preference, mentioned in 15 studies detailed in Table 3, aligns with recent research highlighting the central role of these platforms in organizing content, facilitating academic interaction, and providing secure virtual environments that enable the progressive integration of diverse cultural perspectives ( Machwate et al., 2021; Oudat & Othman, 2024).

The predominance of collaborative methodologies such as COIL (Collaborative Online International Learning) and blended learning in the reviewed studies aligns with previous research emphasizing the pedagogical value of structured virtual interaction as an effective means to foster intercultural competence ( Areskoug et al., 2022; Ayazbayeva et al., 2025). In this regard, experiences documented by Borger (2022), Dai (2021), and Guillén-Yparrea & Ramírez-Montoya (2023a, 2023b) coincide with the findings of Ayazbayeva et al. (2025), who demonstrated that participation in virtual programs and digital intercultural activities during the pandemic significantly contributed to the development of communicative competence and cultural sensitivity among higher education students.

This study confirms a significant relationship between digital competence and the ability to operate in intercultural contexts, especially in educational settings with structural challenges, such as rural areas. These findings align with research by Cabero-Almenara et al. (2023), who found that teachers with higher levels of digital competence also exhibited more effective performance in intercultural situations, suggesting a critical interdependence between these two dimensions. This result reinforces the need to strengthen teacher training in educational technologies from an intercultural perspective. Likewise, Isaeva et al. (2025) emphasize the importance of integrating emerging technologies such as artificial intelligence, gamification, and adaptive platforms to enrich the development of intercultural competences, particularly in contexts characterized by high cultural diversity or low connectivity.

In contrast to the predominance of structured educational technologies such as COIL, blended learning, or LMS platforms in the selected studies, research by Rauer et al. (2021) and Louahala (2023) highlights the inclusion of social networks as a valuable alternative for developing intercultural competences. These studies demonstrate that tools like Facebook, virtual forums, and other social platforms can facilitate more spontaneous, horizontal, and culturally authentic interactions among students from different backgrounds.

Despite this potential, the majority of the analyzed studies do not consider social networks as primary pedagogical resources, indicating limited integration of informal learning spaces. This underutilization has also been noted by authors such as Ngai et al. (2020) and Isaeva et al. (2025), who argue that when appropriately incorporated into instructional design, social platforms can enrich intercultural learning, especially among young audiences accustomed to these digital environments.

Finally, the review reveals a limited presence of studies in non-Western contexts, which constrains understanding of how digital technologies operate in diverse cultural settings. Research such as that by Dooly and Darvin (2022) and Ayazbayeva et al. (2025) underscores the need to adapt digital strategies to the specific cultural, social, and economic conditions of each region. Although structured technologies like LMS and formal methodologies predominate, there remains low incorporation of informal tools such as social networks and mobile applications, despite their high educational potential. Parks (2023) notes that integrating these everyday digital environments can foster a more critical, horizontal, and culturally meaningful pedagogy, especially for students in diverse contexts.

### RQ3. In which areas does research on the integration of digital technologies in the teaching of intercultural competences focus, such as communication skills, cultural attitudes, and technological adoption?

The results revealed that the most frequently addressed area in the integration of digital technologies for teaching intercultural competences in higher education is the development of communication skills, identified in 14 of the 23 studies reviewed. This trend reflects a prevailing understanding of intercultural competence as a practical ability for interaction and negotiation of meanings across cultures, aligning with the views of Parks (2023) and Kulich et al. (2021), who emphasize that the foundation of effective intercultural education lies in linguistic and cultural communication mediated by technology.

This emphasis is also supported by Guillén-Yparrea and Ramírez-Montoya (2023b), who, in their systematic review, state that collaborative virtual environments and international experiences are the most effective settings for fostering intercultural communication skills. In this regard, technologies such as COIL (Collaborative Online International Learning) and hybrid learning programs have proven effective strategies for generating authentic interactions, as confirmed by studies such as those by Guo and Xu (2023) and Rubin et al. (2023), which observed a significant increase in pragmatic competence in digital intercultural contexts.

Technological adoption emerges as a growing area, present in five studies within the corpus ( Guillén-Yparrea & Ramírez-Montoya, 2023a; Dugartsyrenova & Sardegna, 2019). This finding aligns with research by Liebech-Lien (2021) and Ayazbayeva et al. (2025), who demonstrate that perceived usefulness, institutional environment, and teacher training are critical factors for successful technological implementation in culturally diverse contexts. These findings are consistent with those of Vilchez et al. (2023) and Dalle et al. (2024), reinforcing the need to strengthen digital literacy as a pathway to more inclusive and culturally relevant education.

Sociolinguistic and intercultural competences, addressed in three studies, indicate a trend toward more comprehensive approaches that go beyond mere verbal exchange. Das et al. (2025) and Lai et al. (2022) emphasize that the use of adaptive platforms and experiential pedagogical experiences significantly contributes to a deeper and more contextualized understanding of linguistic and cultural diversity in language teaching. This component is crucial for advancing higher education that, beyond teaching foreign languages, prepares students to interact sensitively and competently in global contexts.

Other areas such as teacher training, cultural attitudes, and curriculum internationalization were less frequently addressed but remain highly relevant. For example, Rauer et al. (2021) and Woicolesco et al. (2022) document how digital tools can facilitate the integration of an intercultural approach into internationalized curriculum design—a perspective reinforced by Josefsson et al. (2022), who highlight the potential of virtual exchanges to internationalize curricula beyond physical mobility. Studies such as those by Basantes-Andrade et al. (2024a) and Habib-Allah et al. (2024) stress the need to train teachers to integrate intercultural competence into their daily pedagogical practices, particularly in multilingual and digitally mediated contexts.

Other emerging areas were identified, such as autonomy in digital learning, professional training in healthcare, critical thinking, and technical translation, although these remain underrepresented. These less frequently addressed topics indicate that the field is expanding but still requires greater systematization and analysis within specific disciplines. As Fütterer et al. (2023) and Basantes-Andrade et al. (2024b) point out, there is significant formative potential in the use of mobile technologies and social networks, especially for fostering more horizontal, spontaneous, and culturally situated interactions in informal digital spaces. Moving forward, it will be necessary to adopt interdisciplinary approaches that integrate digital competence, critical pedagogy, and cultural contextualization to advance toward more inclusive, global, and sustainable educational models.

### RQ4. Which intercultural competences are addressed?

The findings of this systematic review reflect a panorama aligned with global trends in the study of technology-mediated intercultural competences, albeit with relevant nuances that enrich the understanding of the field. The high frequency observed in the dimension of adaptation and management coincides with recent studies emphasizing the growing need for flexibility and adjustment on the part of individuals in digitalized multicultural contexts ( Zhang, 2024). While Zhang (2024) underscores the importance of cultural adaptability in virtual environments, reviews such as that of Spencer-Oatey and Dauber (2021) continue to prioritize predominantly face-to-face contexts. This difference suggests a paradigm shift driven by virtuality—a phenomenon intensified during the pandemic, as demonstrated by recent research included in this review ( Jørgensen et al., 2020; Sarmiento et al., 2021). Moreover, studies such as that by Rubtsova et al. (2023) offer significant evidence on the use of immersive technologies for the development of these competences, contrasting with works such as that of Shadiev et al. (2024), which remain focused on more traditional theoretical approaches.

The predominance of intercultural knowledge in the analyzed studies aligns with findings reported by Deardorff (2020), who, in her meta-analysis on intercultural competences in higher education, identified the cognitive component as predominant in 58% of research published in Q1 journals. This trend is further reinforced by recent studies, such as Basantes-Andrade et al. (2024b), which highlight the relevance of cultural awareness and the understanding of social norms as key elements for professional integration in globalized contexts. However, authors such as Chuchón-Vilca and Ayala-Villar (2024) and Kramsch and Zhu (2022) warn of a persistent limitation: many educational programs tend to focus on distant cultures, particularly Anglophone ones, rather than engaging with cultures with which students have everyday interactions, potentially limiting the practical effectiveness of these competences.

Regarding communication skills, the results of this review expand and nuance the findings of Shadiev et al. (2021) and Vande et al. (2022), who position digital communication as the backbone of intercultural development, highlighting the role of tools such as social networks, collaborative platforms, and telecollaboration. In this domain, the methodological innovation of initiatives such as COIL (Collaborative Online International Learning) stands out. According to Guillén-Yparrea and Ramírez-Montoya (2023a), COIL’s effectiveness surpasses that of more traditional virtual mobility programs, such as those reported by Hackett et al. (2023), who documented substantial increases in the development of intercultural competences. This emphasis on experiential methodologies is consistent with the findings of Vargas et al. (2024), who underscore that immersive virtual environments enhance fluency and communicative effectiveness in intercultural learning contexts.

Concerning intercultural attitudes, the findings of this review partially coincide with those reported by Perry and Southwell (2022), who identified a predominance of self-administered questionnaires as the primary strategy for measuring these competences. However, this review reveals more innovative approaches, such as the use of virtual reality by Wiesner-Luna and Burgoa-Godoy (2023), which has been shown to induce empathetic experiences more effectively, validated through objective indicators. Likewise, Dai et al. (2025) highlight how intercultural sensitivity can be significantly enhanced through technologies such as virtual simulations, aligning with the conclusions of Wiesner-Luna and Burgoa-Godoy (2023) regarding the transformative power of virtual reality to generate empathy and cultural understanding.

On the other hand, the dimensions of personal development and innovative leadership are the least explored within the analyzed corpus. Nonetheless, recent studies such as that by Melnyk and Koroban (2024) emphasize the potential of immersive technologies to foster inclusive and creative leadership, particularly in teacher training contexts, opening a promising line of research for the future. This finding is consistent with the proposals of Jørgensen et al. (2020) and Zakharova et al. (2019), who stress the need to cultivate these competences to promote more inclusive, reflective, and transformative educational processes.

The results of this review confirm the growing relevance of digital technologies as strategic allies for developing intercultural competences in higher education and reveal areas that remain insufficiently explored, particularly in more reflective and leadership-oriented dimensions. The challenge persists to expand studies toward less represented cultural contexts, with the aim of building a truly global and equitable vision of intercultural learning.

Although technological categories and frequently addressed intercultural competences were identified, there remains a gap in the literature regarding the specific correlation between particular digital tools and the competences developed. Except for some cases—such as the use of COIL to promote intercultural communication—studies rarely establish a direct relationship between the tool used and the competence achieved. This methodological limitation suggests the need for future research using experimental designs that can assess the differential impact of specific technologies (such as virtual reality, gamification, or social networks) on the development of competences such as empathy, global collaboration, or intercultural critical thinking.

### RQ5. What are the limitations of the research?

The findings reveal patterns of limitations consistent with the international literature on technology-mediated intercultural education; however, this analysis also identifies critical nuances that expand the current understanding of the field.

Methodological limitations and institutional collaborative challenges were the most frequently observed, results that align with recent reviews such as that of Guillén-Yparrea and Ramírez-Montoya (2023b), who warn about the predominance of qualitative approaches without triangulation and the limited use of validated instruments in studies on digital intercultural education. This pattern was also identified in Deardorff’s (2020) meta-analysis, where 62% of studies exhibited internal validity issues. Nevertheless, this analysis detected a higher incidence of non-validated instruments specifically in studies with technological components, suggesting an emerging challenge in this subfield.

This methodological deficit contrasts with recommendations from Jackson and Oguro (2018), who advocate for standardized assessment protocols for digital environments. In this vein, there is reinforced support for adopting frameworks such as the “Intercultural Digital Competence Assessment Framework” proposed by Zarceño et al. (2024), which combines validated quantitative metrics with contextualized qualitative analyses.

A distinctive finding of this review is that institutional collaborative challenges surpassed technological barriers (34.78% vs. 17.39%). This differs from studies such as that of Parks (2023), which identified digital infrastructure as the main barrier. The difference may be explained by the post-pandemic timeframe (2021–2024) of this review, a period during which the proliferation of telecollaboration initiatives intensified structural issues such as institutional disengagement, also documented by De Wit and Altbach (2021). In response to these limitations, proposals such as the “staggered collaboration model” from Rokos et al. (2023) stand out, which suggest mandatory pilot phases and formal inter-institutional commitments.

The coexistence of technological barriers and pedagogical limitations confirms the contradiction between technological mastery and the development of intercultural competences highlighted by Vilchez et al. (2023). The results of this study add that, although teacher ethnocentrism is a less frequently reported barrier, it has a disproportionate impact on the sustainability of international collaboration projects, thus expanding on findings from Spencer-Oatey and Dauber (2021), who focused primarily on student-related barriers.

Regarding sampling bias, a concerning trend was observed toward excluding students from study designs, especially in the Latin American context. This pattern reinforces what was reported by Li (2023), although our analysis indicates a higher regional prevalence (47% vs. 28%), consistent with the diagnosis of Dores et al. (2025). This underscores the need to apply frameworks of geographical equity in intercultural research, as proposed by Milani and Portera (2021).

Finally, pedagogical limitations underscore the urgent need for more comprehensive teacher training. The evidence aligns with studies such as that by Dell’Aquila et al. (2023), who recommend the simultaneous integration of digital and intercultural competences into teacher professional development programs.

### RQ6. What are the future lines of research on intercultural competences?

The results show that 91.3% of the reviewed studies explicitly propose future lines of research, evidencing clear scholarly awareness of persistent challenges and emerging opportunities in the development of technology-mediated intercultural competences. This trend aligns with the findings of Basantes-Andrade et al. (2024a) and Deardorff and Arasaratnam-Smith (2021), who emphasize the need for sustained innovation in the face of rapid technological and socio-cultural changes shaping global educational environments.

The most represented category was Assessment and Metrics (82.61%), reflecting a methodological concern for generating solid empirical evidence and valid tools for measuring intercultural competences. This finding is consistent with observations by Sanz et al. (2023), who highlight the scarcity of validated instruments in digital contexts. Similarly, Brunow and Newman (2020), Guillén-Yparrea and Ramírez-Montoya (2023a), and Peifer et al. (2021) insist on the need for longitudinal studies to evaluate the sustainability of intercultural learning and its long-term impact, particularly in programs such as COIL. In this regard, Lyu’s (2024) recommendation to conduct comparisons between face-to-face and virtual models is also relevant, considering the expansion of hybrid modalities in higher education.

Proposals such as designing instruments to measure intercultural critical thinking and evaluating the effectiveness of digital pedagogical strategies in multicultural contexts are consistent with arguments by Zarceño et al. (2024), who advocate for methodological frameworks that combine quantitative and qualitative approaches to comprehensively assess intercultural competence. Likewise, the importance of analyzing the professional impact of these competences has been emphasized by Kosman et al. (2024), reinforcing the need for ongoing assessment beyond the educational environment.

The second most represented category was Technopedagogical Innovation (60.87%), highlighting the development of immersive environments (21.74%) and the personalization of intercultural learning (17.39%). These lines align with recent research by Wang et al. (2024), Dell’Aquila et al. (2023), and Dai (2021), who demonstrate the potential of technologies such as virtual reality, artificial intelligence, and gamification to simulate complex intercultural experiences and promote experiential learning. In line with this, Wiesner-Luna and Burgoa-Godoy (2023) emphasize the usefulness of immersive environments for fostering intercultural empathy. However, there is limited evaluation of the impact of these technologies in real application contexts ( Savelyeva & Sazonova, 2024), as well as scarce exploration of the use of informal spaces such as social networks, which contrasts with the contributions of Parks (2023), who advocates for leveraging these environments as authentic scenarios for intercultural interaction.

Regarding Teacher Training (30.43%), the results highlight the urgent need to integrate digital and intercultural competences into professional development programs. This need aligns with what Zarceño et al. (2024) have indicated, identifying gaps in teacher preparedness to face the challenges of technology-mediated intercultural teaching. Additionally, Dell’Aquila et al. (2023) show how integrated approaches in teacher training enhance effectiveness in international collaboration contexts. The strengthening of multicultural pedagogical content knowledge (PCK), as proposed by Borger (2022), emerges as a key priority, complementing the call from De Wit and Altbach (2021) and Marginson (2022) to strengthen institutional support to overcome these structural deficiencies.

Concerning Specific Contexts (13.04%), there is recognition of the need to apply intercultural competences in fields such as healthcare and business, consistent with the perspectives of Guo and Xu (2023), who highlight the importance of transferring these skills beyond the academic sphere. Furthermore, research lines focused on the effects of the pandemic on higher education are identified, consistent with Kulich et al. (2021) and their analysis of exacerbated inequalities in post-COVID virtual environments. However, there remains low representation of non-Western contexts in the reviewed studies, contrasting with evidence documented by Honen-Delmar and Rega (2023), who highlight successful intercultural practices in settings such as refugee camps.

Institutional Sustainability (13.04%) emerges as a strategic dimension aimed at constructing models of inclusive internationalization. This vision broadens the traditional focus on international mobility ( Tight, 2022), proposing enduring collaborative networks that include institutions from peripheral regions. Finally, the category of Digital Equity (8.70%) addresses a key structural limitation: the persistent digital divide in contexts with low connectivity. This challenge has been highlighted by Vilchez et al. (2023), who warn that without equitable access conditions, efforts to develop digital intercultural competences could exacerbate existing inequalities. The predominance of formal technologies (such as LMS) in the reviewed studies is questioned by authors like Basantes-Andrade et al. (2024b) and Vande et al. (2022), who propose student-centered approaches and the use of more accessible and meaningful technologies. Likewise, the limited attention to the ethical implications of AI contrasts with the framework proposed by Luckin (2025), who urges critical examination of algorithmic biases in multicultural educational scenarios.

The future research lines analyzed reflect convergence with international trends but also reveal critical gaps in assessment, digital equity, and application in diverse contexts that require greater attention in scientific and educational policy agendas.

## Conclusions

This systematic review demonstrates that intercultural competences mediated by digital technologies represent a dynamic and expanding field of research in higher education, particularly since the impetus generated by the pandemic. The dimensions of adaptation and management, intercultural knowledge, communication skills, and intercultural attitudes emerge as the most frequently addressed, reflecting the need to prepare professionals capable of performing effectively in global and multicultural environments. Nevertheless, significant gaps remain in areas such as innovative leadership and personal development, which deserve greater attention given their potential to transform educational processes toward genuine cultural inclusion.

The findings also reveal a marked geographical concentration in the Americas and Europe, which limits the global scope of the phenomenon. Future research should therefore investigate underrepresented contexts, integrate innovative methodologies such as immersive technologies, and promote longitudinal studies to assess the sustained impact of intercultural learning.

From a quantitative perspective, although structured teaching tools such as COIL, virtual classrooms, and learning management systems are widely represented, more flexible technologies, including social media platforms (27%), are also significant. This indicates a coexistence of formal and informal strategies in the digital mediation of intercultural competences, reflecting the diverse pedagogical approaches found across the reviewed studies.

Overall, this review contributes to a deeper understanding of how digital technologies are consolidating as key instruments for the development of intercultural competences, offering new opportunities to design inclusive and culturally relevant educational experiences in higher education.

## Data Availability

No data associated with this article. Figshare: The role of digital technologies in the development of intercultural competences in higher education: A systematic literature mapping (2005-2024), https://doi.org/10.6084/m9.figshare.c.7886789.v1 ( Basantes-Andrade et al., 2025a). License: CC BY 4.0. Figshare: Database: Digital technologies in the teaching of intercultural competences: systematic literature mapping. https://doi.org/10.6084/m9.figshare.29452745 ( Basantes-Andrade et al., 2025b). License: CC BY 4.0 Figshare: PRISMA 2020 checklist_Article. https://doi.org/10.6084/m9.figshare.29374229 ( Basantes-Andrade et al., 2025c). License: CC BY 4.0 Figshare: Flow diagram of the literature selection process according to PRISMA 2020. https://doi.org/10.6084/m9.figshare.29373242.v1 ( Basantes-Andrade et al., 2025d). License: CC BY 4.0
