# Supplementary material for: Integrating Digital Technologies into the Teaching of Intercultural Competences: A Systematic Literature Mapping
Source: F1000Res. 2025 Dec 1;14:772. Originally published 2025 Aug 6. [Version 3] doi: 10.12688/f1000research.167364.3 (PMC12605581; doi:10.12688/f1000research.167364.3)
Supplement: Peer review history

# Reviewer report: Reviewer response for version 3

**DOI:** 10.5256/f1000research.192487.r437728

**Published:** 2026-01-02

**Authors:** Murod Ismailov

**Affiliations:** 1 University of Tsukuba, Tsukuba, Japan

Dear authors, thank you for the thoughtful and constructive responses. I appreciate the clear revisions and methodological clarifications provided throughout. I can now see that the justifications are coherent and strengthen your manuscript. The explanations regarding PRISMA adaptation, temporal trends, data consistency, and category overlaps are clear and appropriate. I also value the corrections made to percentages, geographical classification, and technological categories. Overall, the revisions enhance transparency and improve the scientific rigor of your study.

Are the rationale for, and objectives of, the Systematic Review clearly stated?

Partly

Is the statistical analysis and its interpretation appropriate?

Partly

If this is a Living Systematic Review, is the ‘living’ method appropriate and is the search schedule clearly defined and justified? (‘Living Systematic Review’ or a variation of this term should be included in the title.)

Not applicable

Are sufficient details of the methods and analysis provided to allow replication by others?

No

Are the conclusions drawn adequately supported by the results presented in the review?

Partly

Reviewer Expertise:

educational psychology, telecollaboration

I confirm that I have read this submission and believe that I have an appropriate level of expertise to confirm that it is of an acceptable scientific standard.

# Reviewer report: Reviewer response for version 3

**DOI:** 10.5256/f1000research.192487.r437727

**Published:** 2025-12-31

**Authors:** Lies Sercu

**Affiliations:** 1 Katholieke Universiteit Leuven, Leuven, Belgium

Thank you for your revisions. My suggestion is to accept the manuscript in its current version.

Are the rationale for, and objectives of, the Systematic Review clearly stated?

Partly

Is the statistical analysis and its interpretation appropriate?

Not applicable

If this is a Living Systematic Review, is the ‘living’ method appropriate and is the search schedule clearly defined and justified? (‘Living Systematic Review’ or a variation of this term should be included in the title.)

Partly

Are sufficient details of the methods and analysis provided to allow replication by others?

Yes

Are the conclusions drawn adequately supported by the results presented in the review?

Yes

Reviewer Expertise:

acquisition of intercultural competence; technology-supported promotion of intercultural competence

I confirm that I have read this submission and believe that I have an appropriate level of expertise to confirm that it is of an acceptable scientific standard.

# Reviewer report: Reviewer response for version 2

**DOI:** 10.5256/f1000research.189102.r424819

**Published:** 2025-11-13

**Authors:** Lies Sercu

**Affiliations:** 1 Katholieke Universiteit Leuven, Leuven, Belgium

1. Scope and Objectives :

 The article presents a systematic literature mapping (SLM) of studies published between 2005 and 2024 on the integration of digital technologies in teaching intercultural competences in higher education. It follows PRISMA 2020 guidelines and includes 23 peer-reviewed, open-access studies in English or Spanish.

 While the scope is clearly defined, the rationale and research questions show some misalignment. The gaps identified in the introduction (e.g., lack of longitudinal studies, ethical implications of immersive tech) are not fully addressed by the six research questions, which focus more on descriptive mapping than critical analysis. There is a need to reframe the rationale and clarify the connection between gaps and questions.

 2. Methodological Rigor

 The study combines PRISMA with the Petersen model, ensuring transparency in selection and classification. However, several methodological limitations weaken its robustness:

- Language bias: Only English and Spanish studies were included, excluding potentially rich literature in French, Chinese, Arabic, etc.
- Open-access restriction: This may have excluded high-quality but paywalled research.
- Lack of interrater reliability reporting: Although multiple reviewers were involved, no reliability coefficients are provided.
- Ambiguity in competence definitions: Terms like “intercultural knowledge” are treated as competences, which may require conceptual clarification.

3. Findings and Analysis

 The article offers a comprehensive overview of:

- Geographic concentration: 78% of studies from Americas and Europe, with Mexico, US, and Spain leading.
- Technologies used: LMS platforms (68.18%), COIL/blended learning (45.45%), social networks (27.27%), productivity tools (18.18%).
- Competence dimensions: Adaptation/management (47.83%), intercultural knowledge, communication skills, attitudes (each ~39%).

However, the analysis remains largely descriptive, lacking deeper synthesis or critical engagement with how specific technologies foster particular competences. For example, the article notes the use of social media but does not explore its pedagogical implications in depth.

 4. Novelty and Contribution

 The article’s main novelty lies in:

- Being the first systematic mapping (not a full SLR) of digital technologies in intercultural competence education.
- Highlighting underexplored areas such as innovative leadership (4.35%) and personal development (8.70%).
- Identifying a post-pandemic paradigm shift toward immersive, hybrid, and AI-enhanced learning environments.
- Emphasizing the coexistence of formal and informal digital strategies, with a call to better integrate social media and mobile apps.

These insights are valuable for shaping future research agendas and policy frameworks, especially in terms of digital equity, teacher training, and assessment innovation.

 5. Limitations and Areas for Improvement

 The article acknowledges several limitations in the reviewed studies:

- Institutional collaboration challenges (34.78%)
- Methodological weaknesses (30.43%)
- Sampling bias (26.09%)
- Technological and communication barriers

Yet, the article itself could improve by:

- Providing examples to clarify abstract claims (e.g., immersive environments, pedagogical technologies).
- Avoiding overstatements about its impact (e.g., claiming to establish a reference framework without sufficient empirical grounding).
- Including more critical reflection on the ethical implications of AI and immersive tech.

Conclusion

 This article makes a timely and relevant contribution to the field of digital intercultural education by mapping current practices and identifying critical gaps. Its novelty lies in its methodological approach and its emphasis on emerging technologies and underrepresented competences. However, to fully realize its potential, the study would benefit from greater conceptual clarity, broader linguistic inclusion, and deeper analytical engagement with the pedagogical implications of digital tools.

 Article that is missing from the review:

 Jin, Y., & Sercu, L. (2025). Approaches to Researching Intercultural Competence in Higher Education: A Review of the Literature (2018–2022) as a Platform for Improving the Design of Future Studies on Technology-Supported Interventions. *Review of Educational Research*, 00346543251353803.

Are the rationale for, and objectives of, the Systematic Review clearly stated?

Partly

Is the statistical analysis and its interpretation appropriate?

Not applicable

If this is a Living Systematic Review, is the ‘living’ method appropriate and is the search schedule clearly defined and justified? (‘Living Systematic Review’ or a variation of this term should be included in the title.)

Partly

Are sufficient details of the methods and analysis provided to allow replication by others?

Yes

Are the conclusions drawn adequately supported by the results presented in the review?

Yes

Reviewer Expertise:

acquisition of intercultural competence; technology-supported promotion of intercultural competence

I confirm that I have read this submission and believe that I have an appropriate level of expertise to confirm that it is of an acceptable scientific standard, however I have significant reservations, as outlined above.

## References

[1] Jin, Y., & Sercu, L. (2025). Approaches to Researching Intercultural Competence in Higher Education: A Review of the Literature (2018–2022) as a Platform for Improving the Design of Future Studies on Technology-Supported Interventions. Review of Educational Research, 00346543251353803.

# Response

**Published:** 2025-11-27

**Authors:** Andrea Basantes-Andrade

**Affiliations:** Universidad Técnica del Norte, Ecuador

Dear reviewer,

 We are truly grateful for the dedication and careful attention you invested in evaluating our manuscript. Your thoughtful observations and constructive recommendations have greatly contributed to strengthening the methodological rigor, improving the clarity of the content, and refining the overall structure of the work.

 Below, we present a comprehensive, point-by-point reply to each of your remarks, detailing the specific modifications that have been made in response.

Responses to Reviewer 4

 We deeply appreciate your valuable feedback.

 Kind regards,

 Andrea Basantes-Andrade

# Reviewer report: Reviewer response for version 2

**DOI:** 10.5256/f1000research.189102.r424817

**Published:** 2025-11-11

**Authors:** Nicia Guillén Yparrea

**Affiliations:** 1 Engineering and Sciences School, Tecnologico de Monterrey, Monterrey, Mexico

Dear Authors,

 I hope you are doing great.

 It was a pleasure to review your manuscript. I share my recommendations in order to improve its quality.

 Evaluation:

 1. Validity of the Study

 The study addresses the timely and relevant topic of integrating digital technologies to develop intercultural competencies in higher education. Its theoretical foundation is solid and grounded in contemporary perspectives on interculturality and digital literacy. Using a systematic literature mapping (SLM) approach is appropriate for identifying trends and research gaps. However, the main research question and comparative scope across regions and disciplines should be clearer. This would strengthen the study's external validity and clarify its specific contribution to the broader field of intercultural digital education.

 2. Data and Methods:

 The methodology is appropriate and described in sufficient detail. The inclusion and exclusion criteria are well-defined, and the analysis process follows the PRISMA 2020 recommendations. To enhance transparency, it would be useful to specify the screening procedure and justify the exclusion of studies in other languages. Including a supplementary table listing all analyzed articles would improve replicability and traceability further. Overall, the methods are rigorous and aligned with the open research standards promoted by F1000Research.

 3. Interpretation:

 The results are well-organized and coherently interpreted. The paper effectively identifies patterns, gaps, and thematic clusters, providing valuable insights to the field. However, the discussion could be enriched by delving deeper into the pedagogical implications of digital tools for intercultural learning and by comparing the findings with those from studies in underrepresented regions, such as Latin America and Africa. Finally, a reflection on how the results can inform curriculum design or internationalization policies would enhance the paper’s applied relevance and theoretical impact.

 4. Clarity and Presentation:

 The manuscript is clearly written and well-structured. The figures and tables are relevant and effectively support the narrative. Minor language editing is recommended to ensure consistency in terminology (e.g., intercultural competence versus digital intercultural literacy). Adding a short "Limitations" section before the conclusions, acknowledging temporal, linguistic, and methodological scope, would improve transparency. 

 Please note that I used "DeepL Write" to improve the writing of this review.

Are the rationale for, and objectives of, the Systematic Review clearly stated?

Partly

Is the statistical analysis and its interpretation appropriate?

Not applicable

If this is a Living Systematic Review, is the ‘living’ method appropriate and is the search schedule clearly defined and justified? (‘Living Systematic Review’ or a variation of this term should be included in the title.)

Not applicable

Are sufficient details of the methods and analysis provided to allow replication by others?

Yes

Are the conclusions drawn adequately supported by the results presented in the review?

Yes

Reviewer Expertise:

Intercultural Education, International Education, Educational Technology, Educational Innovation

I confirm that I have read this submission and believe that I have an appropriate level of expertise to confirm that it is of an acceptable scientific standard, however I have significant reservations, as outlined above.

# Response

**Published:** 2025-11-27

**Authors:** Andrea Basantes-Andrade

**Affiliations:** Universidad Técnica del Norte, Ecuador

Dear reviewer,

 We sincerely appreciate the time and effort dedicated to reviewing our manuscript. Your insightful comments and constructive suggestions have been instrumental in enhancing the scientific rigor, clarity, and overall coherence of the paper.

 Below, we provide a detailed, point-by-point response to each of your comments, indicating the specific revisions that have been incorporated into the manuscript accordingly.

Reviewer 3's Responses

 Thank you again for your valuable review. We remain available for any further comments.

 Best regards,

 Andrea Basantes-Andrade

# Reviewer report: Reviewer response for version 1

**DOI:** 10.5256/f1000research.184474.r407498

**Published:** 2025-09-05

**Authors:** Murod Ismailov

**Affiliations:** 1 University of Tsukuba, Tsukuba, Japan

This is a timely and relevant study that maps research on the integration of digital technologies into intercultural competence teaching in higher education. The topic is important and the dataset is valuable, particularly in highlighting trends since 2019. The use of PRISMA brings transparency to the selection process, and the six research questions are well formulated.

 However, I see several areas where the paper needs clarification and corrections before it can be indexed. These relate mainly to methodological clarity, numerical consistency, and the alignment between results and conclusions. In its current form, some findings may be misleading to readers because of overlapping categories, unclear data handling, and inconsistent reporting.

 Major Issues

 1 - Methodological concerns

 The paper calls itself a “systematic literature mapping” (SLM) but applies PRISMA 2020, which is standard for systematic literature reviews (SLRs). These two approaches have different purposes and levels of rigor. If PRISMA is being used for an SLM, please explain how it was adapted and why it was chosen over mapping-specific frameworks.

 2 - Confusion with coverage period vs. actual results

 While the review covers 2005–2024, nearly 87% of the included studies are from 2019 onward. Was this due to a genuine lack of earlier research, or might the search strategy have favored recent publications? A short explanation would help readers interpret the temporal trends.

 3 - PRISMA flow diagram  promems

 The numbers don’t quite add up. You report 176 initial records and 5 duplicates removed, which should leave 171, but the screening stage starts with 155. Please check and correct the counts so the selection process is transparent.

 4 - Check inclusion criteria consistency

 Inclusion criterion IC5 requires that studies integrate technology into intercultural competence teaching. At least one included study (Ciftci & Savas, 2018) does not clearly meet this requirement. If exceptions are made, they should be explicitly justified.

 5 - Problem with percentage discrepancies

 For example, RQ3 states that “communication skills” is the focus of 42% of studies, but Table 4 lists 14 out of 23 studies (~61%). Please review all reported percentages to ensure they match the underlying data.

 6 - competence dimensions overlap

 In RQ4, many studies appear in multiple categories (e.g., communication skills and intercultural attitudes). This is fine, but the paper should clarify whether percentages are exclusive or overlapping so readers understand how totals are calculated.

 7 - Geographic mis-classification?

 Scotland is listed separately from the United Kingdom, which inflates diversity counts. Unless a study explicitly treats Scotland as separate from the UK, these should be merged for consistency.

 8 - Another overlap - Technology category

 Some tools (e.g., COIL) are counted in more than one category, which can inflate category frequencies. Please define clear, mutually exclusive categories or explain why overlaps are reported.

 9 - Mismatch between your results and conclusions

 The conclusion states there is a “clear preference for structured teaching tools,” but 27% of studies use social media, which is not negligible. This should be discussed so conclusions reflect the actual distribution.

 10 - Peer review compliance issue

 Some included works (e.g., Munoz-Escalona et al., 2020) may not clearly meet the “peer-reviewed” criterion. Please verify the status of each study and remove any that do not meet IC2.

*Other technical / presentation issues. Please:*

1. Clarify why an SLM is using PRISMA and how it was adapted.
2. Ensure all PRISMA counts match the narrative.
3. Recalculate and correct all percentages.
4. State clearly if competence dimension percentages are overlapping or exclusive.
5. Standardize country/region reporting.
6. Double-check that all included studies meet the stated inclusion criteria.
7. Define mutually exclusive technology categories.
8. Add clearer figure/table legends, especially for calculation bases.
9. Include a breakdown of methodological quality scores for each study.
10. Link each conclusion directly to quantitative evidence from the results.

**My recommendation - Major Revision** – The study has some value, but key methodological and reporting issues must be addressed. I would like the authors to correct the numerical inconsistencies /  clarify overlaps, / align conclusions more closely with the data ... these should somewhat strengthen the paper. Good luck.

Are the rationale for, and objectives of, the Systematic Review clearly stated?

Partly

Is the statistical analysis and its interpretation appropriate?

Partly

If this is a Living Systematic Review, is the ‘living’ method appropriate and is the search schedule clearly defined and justified? (‘Living Systematic Review’ or a variation of this term should be included in the title.)

Not applicable

Are sufficient details of the methods and analysis provided to allow replication by others?

No

Are the conclusions drawn adequately supported by the results presented in the review?

Partly

Reviewer Expertise:

telecollaboration, intercultural communication, digital learning

I confirm that I have read this submission and believe that I have an appropriate level of expertise to confirm that it is of an acceptable scientific standard, however I have significant reservations, as outlined above.

# Response

**Published:** 2025-09-23

**Authors:** Andrea Basantes-Andrade

**Affiliations:** Universidad Técnica del Norte, Ecuador

Dear Reviewer,

 We sincerely appreciate the time and effort you have dedicated to evaluating our manuscript. Your comments and suggestions have been extremely valuable in strengthening the scientific quality, clarity, and overall coherence of the work.

 Below, we provide a detailed response to each of your comments, indicating the revisions incorporated into the manuscript and, where applicable, the justification for maintaining the original wording.

https://f1000research-files.f1000.com/linked/754109.Reviewer_2%27s_Responses.docx

 Sincerely,

 PhD. Andrea Basantes

# Reviewer report: Reviewer response for version 1

**DOI:** 10.5256/f1000research.184474.r404966

**Published:** 2025-08-25

**Authors:** Eleni Korosidou

**Affiliations:** 1 University of Western Macedonia, Florina, Greece

This is a well-written study that makes a valuable contribution to understanding the integration of digital technologies in intercultural teaching, offering a solid basis for further research in the field.

 However, the following aspects merit consideration to reinforce the study.

 The inclusion criterion IC1, “Scientific articles written in English or Spanish,” may have influenced the results for RQ1, as the concentration of research in the Americas and Europe could be partly explained by the acceptance of Spanish-language publications. The finding that Mexico leads scientific production with 15% (n=4), followed by the United States, Spain, and Russia with 11% each (n=3), should therefore be acknowledged as potentially shaped by language-based inclusion.

 The inclusion of Ciftci and Savas (2018) also appears problematic, since the study emphasizes the benefits of technologies in intercultural teaching but does not specify the use of particular digital tools, raising questions about consistency with the criteria.

 For RQ3, it remains unclear whether the reported areas of digital technology integration (communication skills, cultural attitudes, and technological adoption) were deliberately targeted in the interventions or whether they emerged as benefits observed during implementation. A similar lack of clarity is noted in RQ4 with respect to intercultural competences: it is not evident whether these were explicitly addressed in the design of interventions or whether they developed incidentally during the research process. Finally, the potential influence of specific digital tools on the development of particular competences is not sufficiently addressed, and further insights in this direction would strengthen the study.

Are the rationale for, and objectives of, the Systematic Review clearly stated?

Yes

Is the statistical analysis and its interpretation appropriate?

Partly

If this is a Living Systematic Review, is the ‘living’ method appropriate and is the search schedule clearly defined and justified? (‘Living Systematic Review’ or a variation of this term should be included in the title.)

Yes

Are sufficient details of the methods and analysis provided to allow replication by others?

Yes

Are the conclusions drawn adequately supported by the results presented in the review?

Yes

Reviewer Expertise:

Intercultural communication skills and strategies, Language Teaching and Learning, ICT integration

I confirm that I have read this submission and believe that I have an appropriate level of expertise to confirm that it is of an acceptable scientific standard.

# Response

**Published:** 2025-09-01

**Authors:** Andrea Basantes-Andrade

**Affiliations:** Universidad Técnica del Norte, Ecuador

**Dear Reviewer,**

 Warm greetings. We sincerely thank you for the time dedicated to reviewing our manuscript entitled *“Integration of digital technologies in the teaching of intercultural competences: systematic literature mapping”*, and for your valuable comments, which have allowed us to strengthen the quality and rigor of the study.

 Below, we provide detailed responses to each of the comments received:

**Comment 1:**

 The inclusion criterion IC1, “Scientific articles written in English or Spanish,” may have influenced the concentration of research in the Americas and Europe, thus affecting the findings for RQ1.

**Response:**

 We agree that the adopted language criterion (IC1) may have partially influenced the results of RQ1. We acknowledge that the inclusion of publications in Spanish may have favored the representation of studies from Latin American contexts, especially from Mexico and South America. This limitation has been added as a critical observation in the discussion section, in order to make transparent the possible linguistic-geographical bias in the analyzed sample.

**Incorporated explanatory text in the discussion section (at the end of RQ1):**

 Additionally, it is important to consider that the inclusion of studies exclusively in English or Spanish may have limited the geographical representativeness of the sample, favoring research originating from Latin America and Spanish-speaking countries. This linguistic bias may have influenced the predominance of Mexico and Spain in the results; therefore, future reviews could expand the language criterion to include other relevant languages, such as French, Chinese, or Arabic, to better reflect geocultural diversity.

**Comment 2:**

 The inclusion of the study by Ciftci and Savas (2018) appears problematic, as it does not specify particular digital tools.

**Response:**

 After reexamining the study by Ciftci and Savas (2018), we agree that, although it addresses intercultural teaching in digitally mediated contexts, it does not specify particular digital technologies, which raises questions about its alignment with criterion IC5. Therefore, we have marked this case as an exception in the results section and have revised its treatment to clearly distinguish it from studies that do report specific digital tools.

**Text added in the article (in RQ2):**

 It is worth noting that the study by Ciftci and Savas (2018) constitutes an exception, as it recognizes the benefits of using technologies in intercultural contexts but does not describe specific digital tools implemented in the intervention. This limits its comparability with other studies in the corpus and should be considered a methodological limitation in the interpretation of the results.

**Comment 3:**

 In RQ3 and RQ4, it is unclear whether the competences and areas were explicit objectives of the interventions or emerged as incidental outcomes.

**Response:**

 We acknowledge that not all studies in the corpus clearly differentiated between competences explicitly targeted in the pedagogical design and those that emerged during the implementation process. This distinction has been added to the corresponding sections, along with a methodological note in the data extraction section, indicating how both types of findings were recorded.

**Text added in the article, in the Methods section (end of 2.4):**

 It is important to note that during data extraction, both competences and focus areas explicitly stated in the objectives or methodological designs of the studies, as well as those that emerged as observed outcomes or later reflections, were recorded. This distinction was maintained in the analysis matrices, although not all studies clearly differentiated between these levels.

**Text added at the end of RQ3:**

 It is important to mention that in several studies, the intercultural competences and areas of digital integration were identified as emerging benefits during the implementation of the interventions rather than as predefined pedagogical goals. This methodological ambiguity may affect the interpretation of the results and highlights the need for future studies with more explicit designs and structured pre-post evaluations.

**Comment 4:**

 The potential influence of specific digital tools on the development of particular competences is not sufficiently addressed.

**Response:**

 We highly value this observation, which has allowed us to enrich the analysis. We have incorporated an additional reflection in the discussion section, addressing the need for deeper investigation into the correspondence between types of digital tools and specific intercultural competences—particularly in teaching contexts mediated by emerging technologies such as immersive environments or social networks.

**Text added in the article, in the Discussion section (after RQ4):**

 Although technological categories and frequently addressed intercultural competences were identified, there remains a gap in the literature regarding the specific correlation between particular digital tools and the competences developed. Except for some cases—such as the use of COIL to promote intercultural communication—studies rarely establish a direct relationship between the tool used and the competence achieved. This methodological limitation suggests the need for future research using experimental designs that can assess the differential impact of specific technologies (such as virtual reality, gamification, or social networks) on the development of competences such as empathy, global collaboration, or intercultural critical thinking.

 Once again, we sincerely thank you for your suggestions, which have significantly enriched the manuscript and helped us to reinforce its conceptual and methodological soundness. We remain attentive to any further comments you may wish to share.

 Sincerely,

**Dr. Andrea Basantes-Andrade**

 On behalf of the author team

 Universidad Técnica del Norte

 avbasantes@utn.edu.ec
